# Xanthelasma

**DOI:** 10.11604/pamj.2016.25.41.10510

**Published:** 2016-09-29

**Authors:** Taha Elghazi, Zouheir Hafidi

**Affiliations:** 1 Université Mohammed V Souissi, Service d’Ophtalmologie A de l’Hôpital des Spécialités, Centre Hospitalier Universitaire, Rabat, Maroc

**Keywords:** Xanthélasma, hypercholstérolémie, chirurgie, xanthelasma, hypercholsterolemy, surgery

## Image en médecine

Il s'agit d'un patient de 74 ans, hypertendu bien équilibré sous traitement, admis pour une prise en charge d'une cataracte de l'œil droit et chez qui l'examen clinique découvre fortuitement des lésions jaunâtres localisées au niveau des paupières supérieures et inférieures des deux yeux, sous cutanés recouverts d'une peau fine, richement vascularisé. Un bilan a été demandé comprenant un dosage de cholestérol total, HDL, LDL et triglycérides dans le sang mettant en évidence une hypercholestérolémie à 4,2 g/L avec des HDL à 0,5 g/l et des LDL à 2,5 g/l. Le xanthélasma représente une tache jaunâtre au niveau des paupières dues à une accumulation de cholestérol et de lipides. Ils prennent la forme d'une bosse ou d'un léger relief se situant sur la partie des paupières près du nez comme les cas de notre patient. Le xanthélasma constitue un type particulier de xanthome. Les xanthomes sont des nodules bénins sous cutanés formés d'une accumulation de cholestérol. Une prédisposition familiale au xanthélasma est retrouvée et lié à une hypercholestérolémie héréditaire. Le traitement du xanthélasma peut être chirurgical ou par laser.

**Figure 1:**
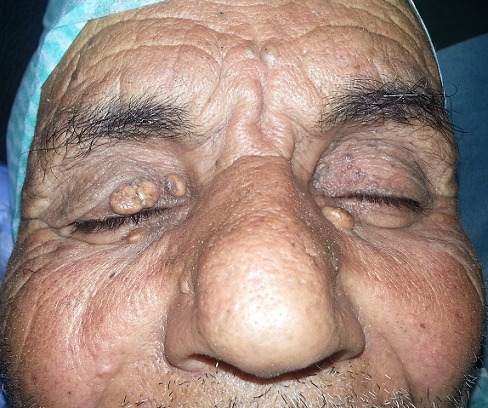
Les différentes lésions jaunâtres au niveau des deux paupières en rapport avec le xanthélasma

